# Recycled Clothing Classification System Using Intelligent IoT and Deep Learning with AlexNet

**DOI:** 10.1155/2021/5544784

**Published:** 2021-03-26

**Authors:** Sun-Kuk Noh

**Affiliations:** National Program of Excellence in Software Center, CHOSUN University, Gwangju, Republic of Korea

## Abstract

Recently, Internet of Things (IoT) and artificial intelligence (AI), led by machine learning and deep learning, have emerged as key technologies of the Fourth Industrial Revolution (4IR). In particular, object recognition technology using deep learning is currently being used in various fields, and thanks to the strong performance and potential of deep learning, many research groups and Information Technology (IT) companies are currently investing heavily in deep learning. The textile industry involves a lot of human resources in all processes, such as raw material collection, dyeing, processing, and sewing, and the wastage of resources and energy and increase in environmental pollution are caused by the short-term waste of clothing produced during these processes. Environmental pollution can be reduced to a great extent through the use of recycled clothing. In Korea, the utilization rate of recycled clothing is increasing, the amount of used clothing is high with the annual consumption being at $56.2 billion, but it is not properly utilized because of the manual recycling clothing collection system. It has several problems such as a closed workplace environment, workers' health, rising labor costs, and low processing speed that make it difficult to apply the existing clothing recognition technology, classified by deformation and overlapping of clothing shapes, when transporting recycled clothing to the conveyor belt. In this study, I propose a recycled clothing classification system with IoT and AI using object recognition technology to the problems. The IoT device consists of Raspberry pi and a camera, and AI uses the transfer-learned AlexNet to classify different types of clothing. As a result of this study, it was confirmed that the types of recycled clothing using artificial intelligence could be predicted and accurate classification work could be performed instead of the experience and know-how of working workers in the clothing classification worksite, which is a closed space. This will lead to the innovative direction of the recycling clothing classification work that was performed by people in the existing working worker. In other words, it is expected that standardization of necessary processes, utilization of artificial intelligence, application of automation system, various cost reduction, and work efficiency improvement will be achieved.

## 1. Introduction

Recently, Internet of Things (IoT) and artificial intelligence (AI), led by machine learning and deep learning, have emerged as key technologies of the Fourth Industrial Revolution (4IR). In particular, object recognition technology using deep learning is currently being used in various fields, and thanks to the strong performance and potential of deep learning, many research groups and Information Technology (IT) companies are currently investing heavily in deep learning [1–6]. As a result, AI is developing at a very rapid pace and will be the core of the IT industry in the future. Computer vision is a research field that examines how computers process images to perform tasks quickly and efficiently. Images collected from cameras are mostly large and have data that are equivalent to human vision. Computer vision has developed simultaneously with computer performance and AI algorithms. Clothing image analysis technology, which has been recently applied to recommend or find specific clothing, includes clothing recognition that analyzes information about clothing images and clothing retrieval, which can help to find desired clothes with images. To apply the deep learning framework, most researchers use convolutional neural networks (CNNs), a popular deep learning technique used to analyze visual imagery [7–10]. Clothing recognition aims to analyze the categories and characteristics of clothes existing in a given image, and networks, such as FashionNet and the Attentive Fashion Grammar Network based on the VGG-16 network, are applied to convolutional neural networks (CNNs). Many studies on CNN-based clothing recognition have focused on clothing classification or clothing detection by processing surveillance camera footage. However, real-time clothing identification from surveillance videos remains a major challenge because of the difficulties involved in achieving reliable clothing detection and representation.

The textile industry involves a lot of human resources in all processes, such as raw material collection, dyeing, processing, and sewing, and the wastage of resources and energy and increase in environmental pollution are caused by the short-term waste of clothing produced during these processes. Environmental pollution can be reduced to a great extent through the use of recycled clothing. Therefore, there have been many efforts in Korea and other countries to recycle and rewear waste clothing. In Korea, the utilization rate of recycled clothing is increasing due to rising awareness of environmental issues and resource conservation [11]. The amount of used clothing is high with the annual consumption being at $56.2 billion, but it is not properly utilized because of the manual recycling clothing collection system, which has several problems such as a closed workplace environment, workers' health, rising labor costs, and low processing speed that make it difficult to apply the existing clothing recognition technology, classified by deformation and overlapping of clothing shapes, when transporting recycled clothing to the conveyor belt. However, recently, the classification method of recycled clothing using mass datasets and deep learning technology has been studied [12, 13].

In this study, I propose a recycled clothing classification system with IoT and AI using object recognition technology to the problems. The IoT device consists of Raspberry pi and a camera, and AI uses the transfer-learned AlexNet to classify different types of clothing. The composition of this thesis is as follows: Section 1 provides Introduction, Section 2 describes related works, Section 3 describes recycled clothing classification system, Section 4 provides measurement results, and Section 5 provides conclusions.

## 2. Related Works

### 2.1. Recycled Clothing

**Figure 1:**
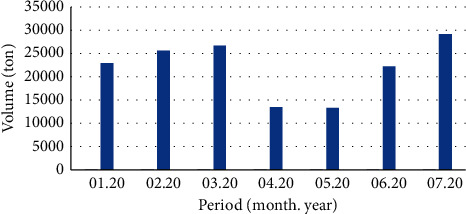
Recycled clothing export in Korea (01.20–07.20).

In Korea, waste clothing collected is classified as recycled and impossible. Reusable clothing is sold in flea markets, bazaars, etc. As shown in Figure 1, recyclable clothing is being exported to foreign countries [14].

### 2.2. Artificial Intelligence and Deep Learning

AI is generally distinguished into two types: machine learning and deep learning. Deep learning is defined as a set of machine learning algorithms that attempt high levels of abstractions through the combination of several nonlinear transformation techniques and is a field of machine learning that integrates people's thinking to computers. Deep learning is a technique that solves problems by stacking neural networks and relies on the amount of data. It makes a structure that flexibly responds to various patterns and cases instead of having few assumptions about problems compared to other machine learning techniques.

### 2.3. Object Recognition and CNN

Object recognition is a computer vision technology that uses computers to identify objects in images. People can easily recognize characters, objects, scenes, and visual details when they see pictures or videos. This ability allows a computer to learn what a person can do with a machine. To solve this problem, deep learning and machine learning algorithms, such as AI technologies, are widely used [15, 16].

Object recognition technology using machine learning is a classification method using machine learning and a CNN and a feature extraction method for image recognition. This method requires a large amount of training data (learning dataset) and requires CNN to set layers and weights. The CNN that has been proposed since 2012 has been using an increasing number of neural layers, and in this process, technologies are used to efficiently insert several neural layers into one CNN. The CNN has a convolutional layer, a pooling layer, and a rectified linear unit (ReLU) as its core elements.

### 2.4. AlexNet

**Figure 2:**
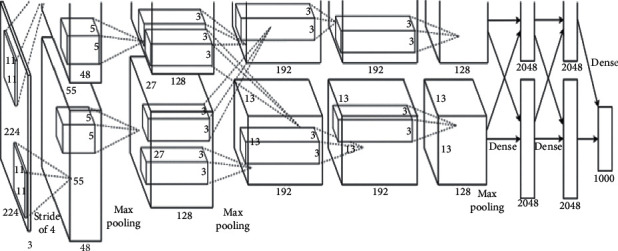
The structure of AlexNet.

AlexNet is a convolutional neural network architecture and consists of eight learning, five convolutional, and three fully connected layers [17–20]. The structure of AlexNet is shown in Figure 2. Usually, the convolutional layers include a convolution step, pooling step, and activation functions. Specifically, the convolution step is a set of learnable filters that are responsible for extracting features from the input image. AlexNet involves 60 million parameters and a neural network with 650,000 neurons consisting of five convolutional layers, some of which consist of three fully connected layers with a maximum pooling layer followed by the last 1000-way softmax. To make training faster, unsaturated neurons and highly efficient Graphic Processing Unit (GPU) convolution operations have been used. To reduce overfitting on fully connected layers, a normalization method called “dropout” was used, and the top test error rate of 15.3% was achieved and compared to the error rate of 26.2% achieved by a variant of this model presented in the ILSVRC 2012 competition. AlexNet has five convolutional layers, three fully connected layers, three max-pooling layers, two cross-channel normalization layers, two dropout layers, and seven rectified linear unit (ReLU) layers. The first layer learns the first-order features, such as color and edges. The second layer learns the corners. The third layer learns about small patches or textures. The fourth and fifth layers learn the higher-order features of the input space. Then, the final-layer features are fed into a supervised layer to complete the task, such as regression. The transfer-learned AlexNet predicts the denoised image from the features learned from the hidden layers.

The structure of AlexNet is shown in Figure 2 that explicitly shows the delineation of responsibilities between the two GPUs. One GPU runs the layer parts at the top of the figure, whereas the other runs the layer parts at the bottom. The GPUs communicate only in certain layers. The network's input is 150,528-dimensional, and the number of neurons in the remaining layers of the network is 290, 400–186, 624–64, 896–64, 896–43, 264–4096–4096–1000.

The convolution neural network AlexNet has five convolution layers (conv), two fully connection layers (fc), and one output layer. The first input image was converted to the size of 227 × 227. The first convolution layer performs the convolution calculation, and in the network, the ReLU activation function is used. The next step is to downsample the feature map, and then the images are normalized. The operation of the second convolution layer is the same as that of the first convolution layer. The third and fourth convolution layers perform only the convolution operation. The fifth convolution layer performs the same operations as the first convolution layer and then inputs the feature map to the fully connected layer. The first fully connected layer and the second fully connected layer are that each neural performs the convolution operation. In the last part of each fully connected layer, the dropout layer should be appended to effectively reduce the number of neurons to prevent overfitting. The final layer is the output layer. It is also a fully connected layer that performs classified work. The neural network uses a loss function to measure the accuracy of image recognition, and then the image recognition uses the tested accuracy to distinguish the image.

### 2.5. Transfer Learning

Transfer learning allows one to reduce the time needed to produce results through the training of models with several images. The calculations of learning methods can be reduced by borrowing from the neural network to solve other problems. This concept is called transfer learning [21, 22]. Transfer learning can overcome certain computational limitations on the use of the deep learning model.

## 3. Recycled Clothing Classification System

### 3.1. Recycled Clothing Classification System

**Figure 3:**
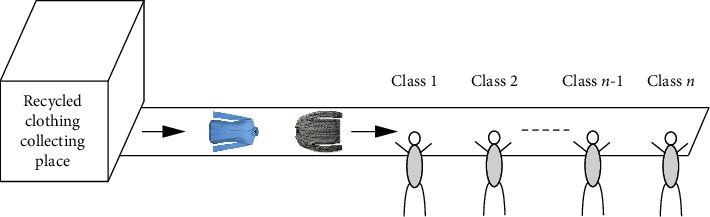
Existing clothing classification system.

**Figure 4:**
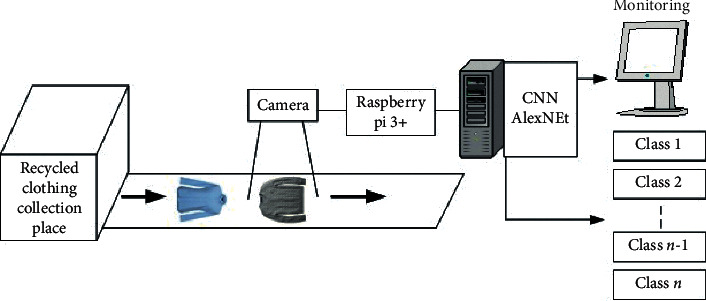
Recycled clothing classification system.

**Figure 5:**
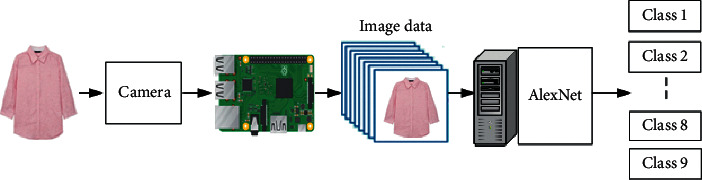
Process of recycled clothing classification using IoT and CNN (AlexNet).

The existing clothing classification system is the same as in Figure 3 in a way that the worker classifies the clothing sent from the recycled clothing collection place (box) in the closed space. This method has problems such as health problems of working workers, various cost increases, and low working speed and efficiency. To solve this problem, a recycled clothing classification system with IoT and AI using object recognition technology was proposed, whose process is shown in Figures 4 and 5. The IoT device consists of a Raspberry Pi and a camera, which captures photographs of recycled clothing, and the image data are transmitted to the AI that uses transfer-learned AlexNet to classify the clothing into nine-class categories, as shown in Figure 5.

### 3.2. Recycled Clothing Classification

**Figure 6:**
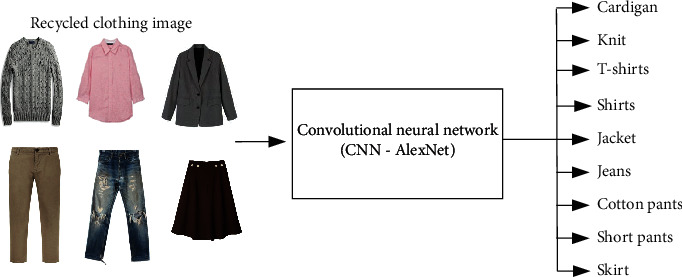
Nine-class clothing classification using CNN.

The details of experimental equipment are listed in Table 1. To classify the clothing images collected by the recycled clothing image datasets, the CNN used transfer-learned AlexNet. AlexNet used in the experiment is a pretrained CNN written in MATLAB, and deep learning to identify the surrounding objects, which is trained on more than 1 million images and can be classified in real time into 1,000 categories, including keyboards, coffee mugs, pencils, and various animals [23]. Using the transfer-learned AlexNet, the recycled clothing image data were categorized into nine groups: cardigans, jackets, shirts, T-shirts, knits, jeans, cotton pants, short pants, and skirts, as shown in Figure 6.

## 4. Measurement and Result

### 4.1. Experimental Environment

**Figure 7:**
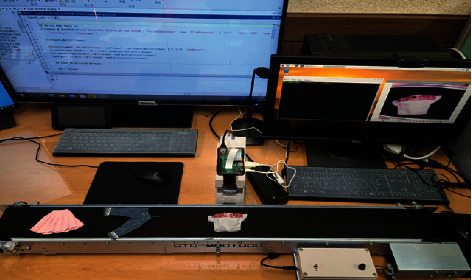
Measurement.

The composition of the experimental equipment is shown in Table 1, and the measurements are shown in Figure 7. The measurement order for the experiment was as follows. The complexity of the calculation time is associated with hardware elements. In this paper, GPU 1EA (NVIDIA GeForce RTX 2080 Ti) is used (Table 1).

First, recycled clothing data were collected from 2400 clean image data without crumpled or folded clothing and 900 loss image data with crumpled or folded clothing. These data (total: 3,300) were used as inputs for AlexNet. To classify clothing images collected by the clothing image datasets, the CNN used transfer-learned AlexNet, written in MATLAB [24].

We trained our model using stochastic gradient descent with a batch size of 64, epochs at 3, and an initial learning rate of 0.001.

### 4.2. Data Collection and Processing

The clothing image data used in the deep learning-based clothing image classification model study were collected by crawling the clothing image existing in Google, as shown in Table 2. The collected images were stored as clean images, and some were removed from clean images and stored as loss images. The loss image was reflected when the clothes placed on the conveyor belt were crumpled or folded at the worksite. Images of different sizes were changed to a uniform size of 257 ⨯ 257.

### 4.3. Model Design and Implementation

The AlexNet model was used as a deep learning-based clothing image classification model, the metastatic learning model was the same as the table, and MATLAB was used. Learning continues in the direction of reducing the cross-entropy loss function, which is the same as defined in the following formula:

(1) $$\begin{array}{c} \text{CE}=-\sum_{i}^{c}t_{i}\log\left(s_{i}\;\right). \end{array}$$

Here, *t*_*i*_ and *s*_*i*_ are the ground truth that is the correct answer, and the last output vector of the model, which is the *i*-th element of the result value of the softmax layer of the model, respectively. To minimize this, learning continues using the SGD algorithm. Finally, the prediction probability of each category is derived through the softmax layer using the image characteristics of the global average pooling layer, and the classification is learned using it. The experimental conditions are the same as shown in Table 3.

For model learning, the classification results were verified with 2400 data for learning and 900 data for testing, with a 7 : 3 ratio of all 3,300 clothing image data. The model was trained with 2310 images in nine classes and tested with 990 test images. The loss function used by the model was half mean squared error. Model visual knowledge depends on the feature activation induced by the last layer. The transfer-learning AlexNet model was used as a deep learning-based clothing image classification model, and the results of classifying the clothing image data taken using IoT as inputs are as follows.

### 4.4. Measurement Results of Classification Experiments

**Figure 8:**
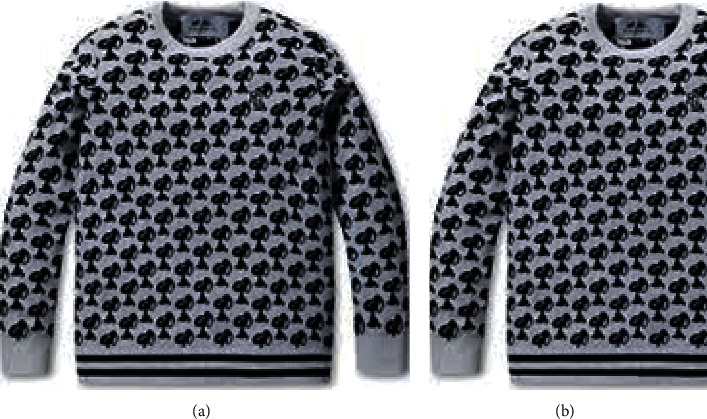
Clean image and loss image. (a) Clean clothing image. (b) Loss clothing image.

**Figure 9:**
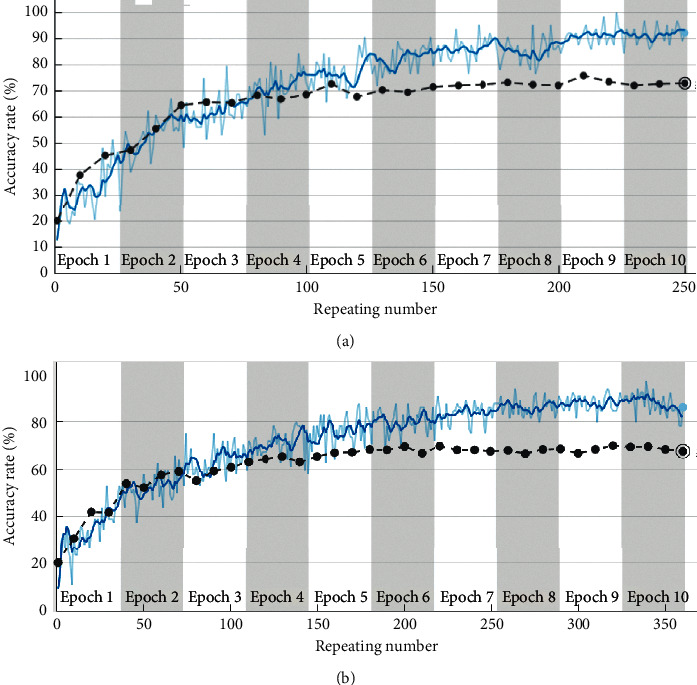
Results of clothing classification using AlexNet. (a) Accuracy of clean image datasets. (b) Accuracy of total image datasets.

**Figure 10:**
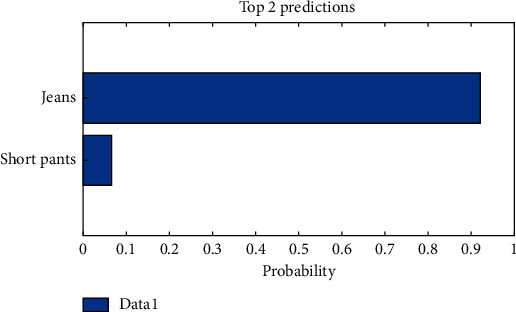
Test clothing image result.

The clean and loss images are shown in Figure 8. The classification results obtained using AlexNet are shown in Figure 9 and Table 4 with the clothing image dataset. This finding confirms that the classification accuracy increases when many clean clothing images are provided to AlexNet as inputs. The costumes consisted of clean images that were perfect images, and loss images, considering transformation and overlap. The clothing image data were classified into nine types: cardigan, jacket, shirt, T-shirt, knit, jeans, cotton pants, short pants, and skirts. The measurement and classification results showed that the classification accuracy of the total clothing images (clean (2400) + loss (900)) at a learning speed of 0.001 and epoch of 10 was approximately 68.28%. The system that classifies images according to the clothing was trained using deep learning, and the clothing classification was demonstrated using the dataset of the clothing image collected by the proposed model. Consequently, clean clothing images were classified with more than 74% accuracy, and total images (loss and clean) were classified with more than 68% accuracy. The test clothing images taken with IoT devices (camera connection to raspberry pies) were classified into the top two of the nine categories using AlexNet deep learning, and the results are shown in Figure 10.

## 5. Conclusions

AI that has been promoted by machine learning and in-depth learning along with new technology to lead 4IR was applied to the real world through various research and developments. With the recent release of GoogLeNet and LesNet's neural networks, the structure of CNN was likely to continue to deepen and become more complicated. Deep learning models were not fixed; therefore, they could be set up in various ways. Hence, if new problems were presented, new neural network models could be developed.

In this paper, we proposed an intelligent classification system for recycled clothing, which classifies them using IoT and AlexNet deep learning. The clothing transferred from the recycled clothing collecting place (box) to the conveyor belt was photographed using a camera connected to Raspberry and an IoT device, and AlexNet deep learning classified the photographed clothing images into nine categories. For the measurement, 3300 clothes image datasets were used.

As a result of this study, it was confirmed that the types of recycled clothing using artificial intelligence could be predicted and accurate classification work could be performed instead of the experience and know-how of working workers in the clothing classification worksite, which is a closed space. This will lead to the innovative direction of the recycling clothing classification work that was performed by people in the existing working worker. In other words, it is expected that standardization of necessary processes, utilization of artificial intelligence, application of automation system, various cost reduction, and work efficiency improvement will be achieved.

## Figures and Tables

**Table 1:** Experimental environment.

IoT device	Raspberry pi 3+
Camera	NOIR 8MP B0153
CNN	AlexNet
GPU	NVIDIA GeForce RTX 2080 Ti
Learning rate	0.001
Epoch	10
Mini batch size	64

**Table 2:** Clothing image datasets.

Class	Clean image	Loss image	Sum image
Cardigans	303	100	403
Cotton pants	200	100	300
Jackets	300	100	400
Jeans	200	100	300
Knits	297	100	397
Shirts	400	100	500
Short pants	200	100	300
Skirts	200	100	300
T-shirts	300	100	400
Total	2400	900	3300

**Table 3:** Experimental equipment.

Layer name	AlexNet model
Conv1	96 number, 11 × 11 × 3 convolution
Conv2	2 groups of 128 5 × 5 × 48 convolution
Conv3	384 number, 3 × 3 × 256 convolution
Conv4	2 groups of 192 3 × 3 × 192 convolution
Conv5	2 groups of 128 3 × 3 × 192 convolution
Pooling	3 × 3 maximum pooling

**Table 4:** Measurement results of classification experiments.

Image	Clothing dataset	Accuracy (%)
Clean	2400	74.20
Loss	900	53.33
Total	3300	68.28

## Data Availability

Previously reported data were used to support this study, and these prior studies are cited at relevant places within the text as reference [20].
